# Who Could Be Blamed in the Case of Discrepant Histology and Serology Results for *Helicobacter pylori* Detection?

**DOI:** 10.3390/diagnostics12010133

**Published:** 2022-01-06

**Authors:** Sabine Skrebinska, Francis Megraud, Ilva Daugule, Daiga Santare, Sergejs Isajevs, Inta Liepniece-Karele, Inga Bogdanova, Dace Rudzite, Reinis Vangravs, Ilze Kikuste, Aigars Vanags, Ivars Tolmanis, Selga Savcenko, Chloé Alix, Rolando Herrero, Jin Young Park, Marcis Leja

**Affiliations:** 1 Institute of Clinical and Preventive Medicine & Faculty of Medicine, University of Latvia, LV-1586 Riga, Latvia; ilva.daugule@lu.lv (I.D.); daiga.santare@lu.lv (D.S.); sergejs.isajevs@lu.lv (S.I.); inta.liepniece-karele@lu.lv (I.L.-K.); inga.bogdanova@lu.lv (I.B.); dacerudzite2008@inbox.lv (D.R.); reinis.vangravs@lu.lv (R.V.); ilze.kikuste@gastrocentrs.lv (I.K.); aigars.vanags@lu.lv (A.V.); selga15@inbox.lv (S.S.); marcis.leja@lu.lv (M.L.); 2 Faculty of Residency, Riga Stradins University, LV-1007 Riga, Latvia; 3 French National Reference Centre for Campylobacters and Helicobacters, Bacteriology Laboratory, Bordeaux University Hospital, 33076 Bordeaux, France; francis.megraud@u-bordeaux.fr (F.M.); chloe.alix8@gmail.com (C.A.); 4 Campus of Carreire, INSERM U1053 BaRITOn, University of Bordeaux, 33000 Bordeaux, France; 5 Riga East University Hospital, LV-1038 Riga, Latvia; 6 Academic Histology Laboratory, LV-1073 Riga, Latvia; 7 Digestive Diseases Centre “GASTRO”, LV-1079 Riga, Latvia; ivars.tolmanis@lu.lv; 8 Agencia Costarricense de Investigaciones Biomedicas, Fundación INCIENSA, San Jose 2250, Costa Rica; HerreroR@iarc.fr; 9 International Agency for Research on Cancer, Early Detection, Prevention and Infections Branch, 69372 Lyon, France; ParkJY@iarc.fr

**Keywords:** *H. pylori*, histology, serology, discrepant cases, polymerase chain reaction

## Abstract

Background. Discrepancies between histology and serology results for *Helicobacter pylori* detection could be caused by a variety of factors, including a biopsy sampling error, expertise of the pathologist, natural loss of infection due to advanced atrophy, or a false-positive serology in the case of a previous infection, since antibodies may be present in blood following recovery from the infection. Aims. To identify true *H. pylori*-positive individuals in discrepant cases by serology and histology using real time polymerase chain reaction (RT-PCR) as a gold standard. Methods. Study subjects with discrepant histology and serology results were selected from the GISTAR pilot study data base in Latvia. Subjects having received previous *H. pylori* eradication therapy or reporting use of proton pump inhibitors, antibacterial medications, or bismuth containing drugs one month prior to upper endoscopy were excluded. We compared the discrepant cases to the corresponding results of RT-PCR performed on gastric biopsies. Results. In total, 97 individuals with discrepant results were identified: 81 subjects were serology-positive/histology-negative, while 16 were serology-negative/histology-positive. Among the serology-positive/histology-negative cases, 64/81 (79.0%) were false-positives by serology and, for the majority, inflammation was absent in all biopsies, while, in the serology-negative/histology-positive group, only 6.2% were proven false-positives by histology. Conclusions. Among this high *H. pylori* prevalent, middle-aged population, the majority of discrepant cases between serology and histology were due to false positive-serology, rather than false-negative histology. This confirms the available evidence that the choice of treatment should not be based solely on the serological results, but also after excluding previous, self-reported eradication therapy.

## 1. Introduction

The role of serology in *Helicobacter pylori* detection has been debated for decades. Already, the *Maastricht II* recommendations have suggested to abandon the use of serology for clinical purposes and to limit its use to epidemiological studies only [1]. Nonetheless, in many countries, it is the most widely used test for *H. pylori* detection today. The most recent *Maastricht V/Florence* guidelines [2] limit the use of serology to specific cases, for example, in atrophy, considering that the bacterial load could be significantly decreased in severe gastric mucosal atrophy. This was effectively demonstrated by Kokkola et al. in a study where there were significantly decreased *H. pylori* antibody titers in individuals with atrophic gastritis with negative histology and ^13^C-urea breath test after prescribing an eradication treatment [3]. Theoretically, under these circumstances, serology might indicate the true status of infection, while histology might fail to identify the presence of the bacterium [4]. However, advanced gastric atrophy and the total disappearance of *H. pylori* results in a false positive serology, since antibodies can be found for a prolonged period (up to five years), making it impossible to distinguish active from past infection [5,6].

Serology is still widely recommended in Asia as one of several noninvasive methods for mass screening strategies in populations with increased risk of gastric cancer [7]. The ABC (D) method proposed by Miki [8] combines the noninvasive detection of atrophy by pepsinogen detection with *H. pylori* detection based on serology. Contrary to the approach in Europe, testing with the ABC method (including *H. pylori* serology as a part of it) has been introduced in some gastric cancer primary screening settings in Japan and could be an effective tool for gastric cancer screening [9]. It is noteworthy that panel testing for pepsinogens and *H. pylori* serology (with or without gastrin-17 detection) has gained significant interest and applicability also in Europe during the last decade [10]. Therefore, in fact, the real clinical practice in Europe is significantly contradicting what is recommended in the European guidelines.

The German guidelines proposed probably the most pragmatic approach, by recommending that serological assessment of *H. pylori* could be used, while any positive test results have to be confirmed with another, more reliable, test (such as ^13^C-urea breath test, stool antigen test, or invasive methods such as histology, culture, or rapid urease test) prior to the prescription of an eradication therapy [11].

Histological analysis of *H. pylori* has been considered as the ‘*gold standard*’; however, certain limitations also apply for this method, and it can give imprecise results under certain conditions as well. Theoretically, a patchy distribution of *H. pylori* infection, technical issues including lack of specific staining, low microscope quality, as well as the insufficient expertise of the pathologists may lead to false negative results in histological analysis. The patchy distribution of bacteria was demonstrated in a study by Pichon et al. [12], analyzing 365 gastric biopsies from the stomachs of three patients after gastrectomy and showing that *H. pylori* was missed in 3% (11/365) of cultured biopsies. However, most importantly, a very low bacterial density resulting from severe atrophy or proton pump inhibitor (PPI) intake may significantly alter the detection of *H. pylori* microscopically. Some of the previous recommendations from expert pathologists in the field recommended to also include in the report the intensity of neutrophil infiltration, characterizing an active inflammation, particularly in PPI users.

Another important issue is the false-positive results of the histological assessment of *H. pylori* due to misinterpretation of other urease-containing bacteria than *H. pylori*, as published by Osaki et al. [13]. This could be diminished by using immunohistochemistry (IHC) in addition to Giemsa staining, since immunohistochemical staining with specific *H. pylori* antibodies shows the highest specificity for the detection of *H. pylori*. [14]. However, IHC is recommended only in individuals with active gastritis without *H. pylori* identification by histochemistry. To summarize, there are reasons why the ‘*gold standard*’ may still appear to be imperfect.

Thus, despite advances in *H. pylori* diagnostics, drawbacks for each method still exist, and it is important to select the best method for a *H. pylori* search and treat strategy, as well as for the diagnosis of infection in individuals with gastric atrophy, bleeding, or other medical conditions. Since serology is often used in individuals over 50 with a higher probability of having atrophic changes, it is important to identify whether histology or serology is more accurate.

Recently, a different molecular method for *H. pylori* identification, real-time PCR (RT-PCR), was developed and, at present, it is considered to be highly precise [15]. Detection of *H. pylori* by molecular biology methods could identify the presence of bacteria in culture-negative biopsies, as demonstrated by Pichon et al. [16]. Therefore, in doubtful cases, it could be used to confirm *H. pylori* diagnosis.

The primary objective was to identify true positive results in doubtful cases with RT-PCR, used arbitrarily as the gold standard, and the secondary objective was to investigate the reasons for discrepancies between serology and histology results.

## 2. Material and Methods

### 2.1. Study Design

The study was conducted using data from the gastric cancer prevention study by predicting atrophic gastritis (GISTAR) pilot study (http://www.gistar.eu (accessed on 10 December 2021)). The details of the pilot study [17] and the general study design are available elsewhere [18].

A standardized and quality-controlled approach to serology and pathology according to the GISTAR protocol allowed discrepancy issues to be addressed in a reliable way by selecting cases with different *H. pylori* statuses according to the serology and histology results from participants having undergone upper endoscopies. Furthermore, biopsy samples were analyzed with RT-PCR to identify the true *H. pylori* infection status.

### 2.2. Participants

Asymptomatic individuals (aged 40–64 years at inclusion), who had undergone upper digestive endoscopy according to the study protocol and had discrepancies between *H. pylori* serology and histology results were included in the analysis.

**Figure 1:**
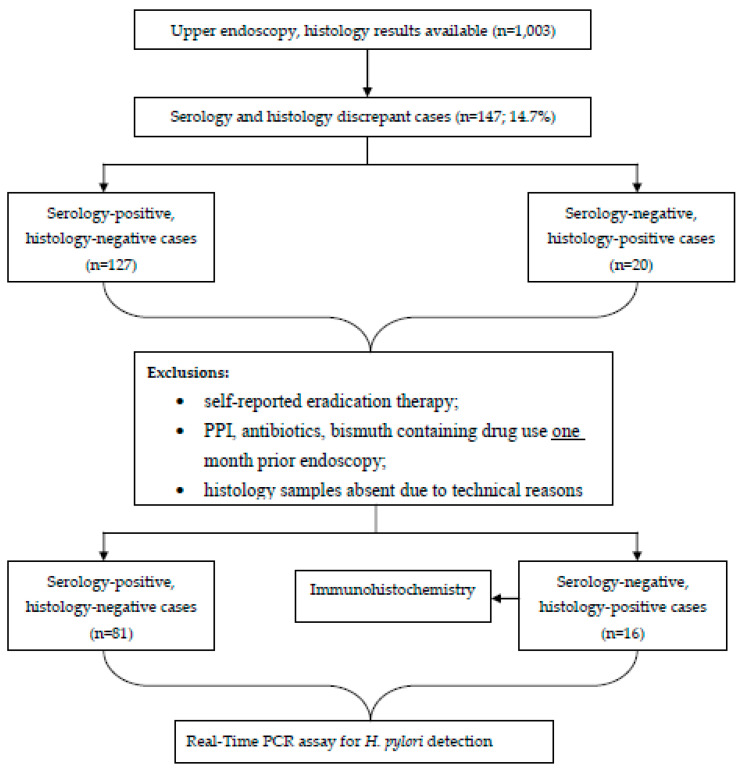
Flow chart of the study design.

Individuals having self-reported *H. pylori* eradication therapy at any time-point or who reported the use of PPI, antibacterial medications, or bismuth-containing drugs one month prior to endoscopy were excluded, as recommended by the latest *Maastricht* guidelines [2]. Study design in flow-chart is shown in Figure 1.

### 2.3. Serology

Blood samples were collected according to a study protocol described in detail elsewhere [17,18].

Immunoglobulin G group antibodies to *H. pylori* infection (*Helicobacter pylori IgG ELISA*, Biohit Oyj, Helsinki, Finland) were measured in plasma using an automated ELISA instrument (Personal LAB., Adaltis, Guidonia Montecelio, Italy). The cut-off value for antibody positivity was ≥30.0 EUI. The sensitivity and specificity were 93.3% and 95.7%, respectively [19].

Pepsinogen levels (PgI/II) were detected by an ELISA system, the Gastropanel^®^ (Biohit Oyj). Based on the manufacturer’s cut-off values, a decreased pepsinogen level was considered regarding whether the PgI/PgII ratio was ≤3.0 or the PgI was ≤30 ng/mL.

### 2.4. Histology

Five non-targeted (random) biopsy specimens for histology were obtained according to the updated Sydney system [20], stored in 10% formalin in separate vials (marked accordingly). From the paraffin-embedded biopsies, 3 μm thick sections were prepared and stained with hematoxylin and eosin and modified Giemsa stain.

Two gastrointestinal pathologists assessed the samples independently, and a consensus was used for the current analysis. The *H. pylori* detection was reported as positive if any of the biopsies provided a positive result for the presence of *H. pylori* type bacteria.

The presence of gastric atrophy was evaluated according to the Operative Link for Gastritis Assessment (OLGA) and Operative Link on Gastric Intestinal Metaplasia Assessment (OLGIM) systems [21]. Inflammation of gastric mucosa was evaluated by the presence of neutrophils. Inflammation was noted if a moderate presence of neutrophils was shown in at least one of five biopsy samples; conversely, it was considered absent if neutrophils were few or not detected in all biopsies.

### 2.5. Immunohistochemistry for H. pylori

IHC was performed only for RT-PCR positive samples to rule out false negative or false positive *H. pylori.* For the IHC examination, *H. pylori* was detected using FLEX, a polyclonal rabbit anti-*Helicobacter pylori* kit (Dako, Glostrup, Denmark). The slides were stained with an automated immunohistochemical technique using automated Ventana, Benchmark Ultra stainer (module; 311969).

The *H. pylori* detection was positive if the apical staining of gastric epithelium cell membranes was present.

### 2.6. Patient Grouping

The study groups were characterized in respect to the presence/absence of inflammation of gastric mucosa histologically and for the presence or absence of atrophy, namely based on OLGA and OLGIM staging systems and pepsinogen detection. Patients were divided into two groups without severe gastric atrophy (i.e., OLGA stage 0, I, or II) and with atrophy (i.e., OLGA stage III, IV, or OLGIM stage III, IV).

### 2.7. Molecular Biology Testing for H. pylori

*H. pylori* deoxyribonucleic acid detection in fresh frozen antral biopsy samples obtained from the major curvature was performed using an RT-PCR in Bordeaux, France. Samples were immediately transferred to sterile 2 mL vials, which contained a specific medium (25 g/L bovine serum albumin (BSA), 74 g/L saccharose, 3.7 mM KH_2_PO_4_, 6.9 mM K_2_HPO_4_, 3.6 mM sodium glutamate and water) to protect *H. pylori* and then stored at −80 °C. Biopsies were shipped to France using an international courier delivery company.

*H. pylori* DNA was isolated by using a QIAamp DNA mini kit (Qiagen SA, Courtaboeuf, France), with glyceraldehyde-3-phosphate dehydrogenase (GAPDH) as the control.

The RT-PCR was performed according to the method developed by Oleastro et al. [22].

### 2.8. Statistics

Statistical analysis was carried out using Microsoft Office Excel 2007, and a statistical package for the social sciences—IBM SPSS Statistics version 22.0.

Frequency distributions were evaluated for the categorical variables (RT-PCR and IHC results, inflammation presence, decreased pepsinogen levels, and gastric atrophy stage) in each group.

### 2.9. Ethical Approval

The GISTAR study protocol was approved by the Ethics Committee of the International Agency for Research on Cancer (IARC; reg. No. IEC 12-36) and the Central Medical Ethics Committee of Latvia.

The current study protocol was approved by the Committee on Ethics of Riga East Clinical University Hospital Support Foundation in Medical and Biomedical Research.

The study protocol is in agreement with the ethical guidelines of the 1975 Declaration of Helsinki. All study participants were required to provide a signed consent prior to the enrolment.

## 3. Results and Discussion

Overall, 147 (or 14.7%) out of 1003 study participants showed discrepant results between histology and serology with respect to *H. pylori* status. According to exclusion criteria, 97 samples were analyzed: 81 were serology-positive/histology-negative, while 16 were serology-negative/histology-positive.

Among serology-positive/histology-negative cases, 79.0% (64/81) were false positives by serology and 21.0% (17/81) were false negatives by histology, using RT-PCR as the gold standard. In the latter group, almost all RT-PCR positive individuals had no inflammation (16/17) in gastric biopsies, while only three had signs of gastric atrophy (OLGA/OLGIM stage III or IV) (Table 1).

At the same time, gastric atrophy was present in 16/64 of the serology-positive/histology-negative and RT-PCR-negative individuals. On the other hand, in PCR-positive cases, almost all had a positive IHC result (15/17) apart from the two samples which were excluded due to technical reasons. The majority of the decreased pepsinogen I/II ratio levels were found in the first group with PCR-negative results for 16 out of 64. In this group, only four subjects (4/64) had gastric atrophy (by the OLGA staging system) and three had intestinal metaplasia (by the OLGIM staging system).

Among **serology-negative/histology-positive** cases, only one (or 6.2%) was a false positive by histology, while 93.8% (15/16) were false negatives by serology.

To specify, among RT-PCR positive individuals, 9 of 15 (60%) had gastric inflammation in at least one biopsy. In addition, all **serology-negative/histology-positive** individuals were IHC positive.

We are reporting here a study providing conclusive evidence on the frequently discussed issue of which method, i.e., histology or serology, is more reliable for the detection of current *H. pylori*. The obtained results show that, in the hands of an experienced pathologist, the pathology provides more reliable results than the serology. It must be noted that the study was possible due to the large number of individuals investigated in the GISTAR cohort, from which a sufficient number of discrepant cases could be selected.

In addition, the study has provided clear evidence on what was previously understood but has been frequently forgotten more recently in clinical practice, namely that the choice of the treatment should not be based solely on serology results, even if the particular individual has denied receiving previous *H. pylori* eradication treatment. In our study, 6.4% (n = 64/1003) of those who were *H. pylori*-positive according to serology were, in fact, negative for an active *H. pylori* infection, although they believed they had never received eradication therapy. This could be explained either by the fact that they did not recall receiving an eradication treatment, or by a parallel *H. pylori* elimination which occurred while being treated with antibiotics for another disease. It would not be acceptable to treat this high proportion of individuals who have no active infection with antibiotics based on serology. This is especially true in the 21st century, when an increasing burden of disease appears related to resistant bacteria. Therefore, we suggest that, in all cases, serological screening for *H. pylori* be confirmed with a UBT, a fecal antigen test, or an endoscopy-based test in case of positivity. This also applies to the test use in the ABC (D) method. Furthermore, serology should not be used in clinical patient management, except in very specific exceptional cases.

In the present study, based on the RT-PCR data used as reference, we tried to detect whether false serology or false pathology was responsible for an imprecise identification of *H. pylori*. Among this middle-aged population with a high *H. pylori* prevalence, the majority of the discrepant cases between serology and histology were due to false-positive serology, rather than false-negative histology.

In total, a discrepancy between serology and histology was observed in 14.7% of cases in our study sample. In a study by Kim et al. [23], discrepant results were observed in 18% (158/872) of subjects with gastric abnormalities (a structural or color change), and, similar to our study, the majority (81/97) were serology-positive–histology-negative. The authors also concluded that gastric adenocarcinoma/adenoma and low serum pepsinogen levels were independent risk factors for a negative histology in serology-positive subjects. Interestingly, follow-up studies with Giemsa staining at different sites of the stomach revealed that 75% of the serology-positive/histology-negative subjects with adenocarcinoma were *H. pylori* positive, while none of those with low serum levels revealed positive findings [23]. In our study, individuals with adenocarcinoma were already excluded from prior analyses, while gastric atrophy could be one of the reasons for discrepancy between serology and histology results.

According to RT-PCR results, false positive serology was responsible for the majority (64/81) of discrepant cases. One of the explanations for serology-positive/histology-negative results could be a previously unreported infection or an infection not known by the study subjects (including *H. pylori* eradication as a result of antibiotic use for another purpose). Although we cannot completely rule out such cases, the interviewers were trained to ask about previous *H. pylori* eradication and any kind of drugs received (especially, antibiotics and PPI).

Furthermore, nonvalidated serological tests could be a cause of false positive serology. However, we used a highly accurate serological test (even the best among several tests), based on a large study by Burucoa et al. [19]. Moreover, the test was also validated in our study population [18]. However, it seems that even validated serology can encounter problems.

The majority of individuals with decreased pepsinogen levels were in the serology-positive/histology-negative group (19/21), while only two were in the serology-negative/histology-positive group. Similarly, all patients with atrophy were in the serology-positive/histology-negative group. Yet, the proportion of individuals with signs of atrophy (serological or histological) was small.

These data point to a possible loss of *H. pylori* due to gastric atrophy, which is also supported by a study led by Kim et al. [23]. Therefore, we can speculate that low serum pepsinogen levels might predict gastric mucosal atrophy, especially in *H. pylori* serology-positive cases. Accordingly, initial gastric atrophy could be one of the reasons for positive serology and negative histology in our sample.

Finally, false-positive results of histological assessment of *H. pylori* could also be considered, due to other urease-positive bacteria. Osaki et al. reported that several bacteria—*Citrobacter freundii, Enterobacter cloacae, Klebsiella pneumoniae, Proteus mirabilis, Staphylococcus aureus* [13] could be misinterpreted as *H. pylori*. Recently, South Korean scientists, using Giemsa stain, noted that *Campylobacter hyointestinalis* could also lead to false-positive *H. pylori* results [24]. However, in these cases, neutrophil activity is the essential and chronic inflammation is less present compared to *H. pylori* infection [24]. The only serology-negative/histology-positive but RT-PCR and IHC negative case could possibly be explained by the presence of *Campylobacters*.

Nevertheless, discrepant cases with positive histology but negative serology could be explained by an imperfect sensitivity of serology, rather than the potential presence of Helicobacters other than *H. pylori*, according to positive IHC analyses in the RT-PCR positive group.

Maastricht V guidelines have nevertheless considered that validated serology could be used for the search and treat strategy, since it is cheaper than UBT and not influenced by previously received treatment [2]. However, prescription of *H. pylori* eradication treatment based on the results of serology could lead to a high and unnecessary use of antibiotics. Therefore, based on our previous results from the pilot study, the main GISTAR study abandoned serology and switched to the use of UBT for *H. pylori* diagnosis [17].

In summary, our study confirms that serology should not be the first choice as a diagnostic tool for the prescription of *H. pylori* eradication treatment.

## 4. Conclusions

Among this middle-aged population with high *H. pylori* prevalence, the majority of discrepant cases between serology and histology were due to false-positive serology cases rather than false-negative histology cases.

Discrepant cases with positive histology but negative serology could be explained by an imperfect sensitivity of serology, more than the potential presence of Helicobacters other than *H. pylori*, according to positive IHC analyses in the RT-PCR positive group.

## Figures and Tables

**Table 1:** Analysis for the total patient sample in *Helicobacter pylori* test discrepancy cases.

HP Status	PCR Result N (%)	IHC Positive N (%)	Inflammation Present in Any of Biopsies N (%)	Inflammation Absent in All Biopsies N (%)	Decreased Pepsinogen N (%)	OLGA III/IV or OLGIM III/IV N (%)
Serology-positive, histology-negative (N = 81)	PCR positive 17 (21.0)	15 ^†^ (88.2)	1 (5.9)	16 (94.1)	3 (17.6)	3 (17.6)
	PCR negative 64 (79.0)	*not performed*	1 (1.6)	63 (98.4)	16 (25.0)	4 (6.3)
Serology-negative, histology-positive (N = 16)	PCR positive 15 (93.8)	15 (100.0)	9 (60.0)	6 (40.0)	1 (6.7)	0
	PCR negative 1 (6.2)	0	0	1 (100.0)	1 (100.0)	0
Overall	PCR positive 32 (33.0)	30 ^†^ (93.5)	10 (31.2)	22 (68.8)	4 (12.5)	3 (9.4)
	PCR negative 65 (67.0)	*not performed*	1 (1.5)	64 (98.5)	17 (26.2)	4 (6.2)

^†^—due to technical reasons, two were missing from the analysis.
